# Secondary prevention strategies after an acute ST-segment elevation myocardial infarction in the *AMI code* era: beyond myocardial mechanical reperfusion

**DOI:** 10.1186/s12872-017-0493-6

**Published:** 2017-02-07

**Authors:** Núria Ribas, Cosme García-García, Oona Meroño, Lluís Recasens, Silvia Pérez-Fernández, Víctor Bazán, Neus Salvatella, Julio Martí-Almor, Jordi Bruguera, Roberto Elosua

**Affiliations:** 1 Cardiology Department, Hospital del Mar, Passeig Marítim, 25-29, 08003 Barcelona, Spain; 2 Heart Diseases Biomedical Research Group, IMIM (Hospital del Mar Medical Research Institute), Barcelona, Spain; 3 Medicine Department, Program in Internal Medicine, Universitat Autònoma de Barcelona, Barcelona, Spain; 4 Hospital Universitari Germans Trias i Pujol, Badalona, Spain; 5 IMIM (Hospital del Mar Medical Research Institute). Cardiovascular Epidemiology and Genetics Group (EGEC), REGICOR Study Group, Barcelona, Spain; 6 CIBER de Enfermedades Cardiovasculares (CIBERCV), Barcelona, Spain

**Keywords:** ST-segment elevation myocardial infarction, Coronary angioplasty, Secondary prevention, Prognosis, Reperfusion therapy, Cardiovascular risk factors

## Abstract

**Background:**

The *AMI code* is a regional network enhancing a rapid and widespread access to reperfusion therapy (giving priority to primary angioplasty) in patients with acute ST-segment elevation myocardial infarction (STEMI). We aimed to assess the long-term control of conventional cardiovascular risk factors after a STEMI among patients included in the *AMI code* registry.

**Design and methods:**

Four hundred and fifty-four patients were prospectively included between June-2009 and April-2013. Clinical characteristics were collected at baseline. The long-term control of cardiovascular risk factors and cardiovascular morbidity/mortality was assessed among the 6-months survivors.

**Results:**

A total of 423 patients overcame the first 6 months after the STEMI episode, of whom 370 (87%) underwent reperfusion therapy (363, 98% of them, with primary angioplasty). At 1-year follow-up, only 263 (62%) had adequate blood pressure control, 123 (29%) had LDL-cholesterol within targeted levels, 126/210 (60%) smokers had withdrawn from their habit and 40/112 (36%) diabetic patients had adequate glycosylated hemoglobin levels. During a median follow-up of 20 (11–30) months, cumulative mortality of 6 month-survivors was 6.1%, with 9.9% of hospital cardiovascular readmissions. The lack of assessment of LDL and HDL-cholesterol were significantly associated with higher mortality and cardiovascular readmission rates.

**Conclusions:**

Whereas implementation of the *AMI code* resulted in a widespread access to rapid reperfusion therapy, its long-term therapeutic benefit may be partially counterbalanced by a manifestly suboptimal control of cardiovascular risk factors. Further efforts should be devoted to secondary prevention strategies after STEMI.

**Electronic supplementary material:**

The online version of this article (doi:10.1186/s12872-017-0493-6) contains supplementary material, which is available to authorized users.

## Background

Case-fatality and long-term mortality after an acute myocardial infarction (AMI) has manifestly decreased over the last decades. Both the use of more effective pharmacological and revascularization strategies account for this decrease [1–4]. However, coronary artery disease (CAD) continues to be the most determinant cause of mortality in high-income societies [5]. ST-segment elevation myocardial infarction (STEMI) is one of the main clinical manifestations of CAD, and rapid (<12 h) coronary reperfusion continuous to be the main therapeutic goal to enhance a favorable cardiovascular outcome after the index coronary event. In this scenario, primary percutaneous coronary intervention (PCI) is nowadays preferred over fibrinolysis, especially when the coronary intervention is performed within the first 120 min [6–8].

Regardless of such rapid therapeutic interventions, the readmission and mortality rates after the index AMI episode are not negligible, with an 8–20% of the patients being readmitted and 10% of them dying within the first year [9–13]. Previous reports have postulated that the suboptimal cardiovascular morbidity and mortality rates after an acute coronary event may be related to an inadequate control of the cardiovascular risk factors (secondary prevention strategies) [14–17]. However, assessment of the long-term degree of implementation and adherence to such secondary prevention strategies in STEMI patients undergoing urgent reperfusion in the era of widespread use of PCI is scarce in the literature [18–21].

In this study, we aimed to assess the long-term control of conventional cardiovascular risk factors in patients presenting with acute STEMI undergoing urgent reperfusion therapy, with urgent PCI designated as the preferred therapeutic strategy. We further analyzed the prognostic impact of an inadequate control of such risk factors.

## Methods

### Patient population: the *AMI code* program

A total of 467 consecutive STEMI patients referred to our hospital from June 2009 to April 2013 within the *AMI code* program were prospectively considered for inclusion. The *AMI code* program, created in 2009, consists of a regional network developed in Catalonia (Spain) aiming to provide a rapid and widespread access to PCI among patients presenting with an acute STEMI [8]. More precisely, it consists of an integrated assistance network for urgent action that coordinates primary health centers, ‘non-PCI-available’ hospitals, ‘PCI-available’ hospitals and mobile emergency assistance units. Upon the diagnosis of STEMI, the *AMI code* is activated and primary PCI (to be performed in a preliminary designated ‘PCI-available’ institution) is set up, provided that the time interval between the onset of symptoms and the percutaneous intervention is presumed to be within the therapeutic window. Additionally, the *AMI code* can be activated for those patients without apparent myocardial reperfusion after pharmacological fibrinolysis (rescue angioplasty). Twenty-four to forty-eight hours after the index STEMI episode, in the absence of acute complications, patients are planned to be discharged to their reference hospital. Patients not established in Catalonia (tourists and displaced) were excluded from the analysis upon a presumably suboptimal follow-up.

The study was designed and implemented in accordance with Guidelines for Good Clinical Practice and with the ethical principles laid down in the Declaration of Helsinki.

### In-hospital data collection

Demographic (age, gender) and clinical characteristics (infarct location, systolic blood pressure, Killip class at admission and left ventricular ejection fraction), as well as history and control of cardiovascular risk factors (smoking, dyslipidemia, hypertension and diabetes mellitus) and other comorbidities (personal and family history of ischemic cardiomyopathy, chronic kidney disease, anemia) were collected at the time of patient’s hospital admission during the index STEMI episode. The lipid profile, glomerular filtration rate and the glycosylated hemoglobin levels were analyzed at baseline. In-hospital morbidity and mortality, the pharmacological therapy administered during admission and the primary PCI procedure findings were also collected.

### Clinical follow-up

The clinical follow-up after final hospital discharge was carried out by the primary care physician or through the cardiologist outpatient clinic/primary health center at the reference physician’s discretion. After hospital discharge, assessment and control of the cardiovascular risk factors and the patient’s clinical outcome were monitored and incorporated into the study database through the Catalonia’s regional electronic health recording system, in which the patient’s clinical status, the rate of hospital readmissions after the index STEMI event, the pharmacological intake, the laboratory test and radiology results were available for the study purposes. Overall mortality was ensured by means of consultation of the National Death Registry. Heart failure, acute coronary syndrome and stroke were considered cardiovascular readmission. Following previously reported definitions, stroke was considered a cardiovascular condition in the assessment of the clinical outcome after STEMI [22–24]. Direct telephone contact with the patient was allowed in order to fulfill any missing data from the electronic health recording system.

The long-term control of conventional cardiovascular risk factors was exclusively analyzed for those patients who survived at least the first 6 months after the index STEMI episode. By these means, any bias in the results obtained related to a suboptimal risk factor control during the very initial phases after the STEMI would be avoided. A minimum 6-months follow-up period was considered compelling for the surviving study patients in order to enhance a truly long-term assessment of the efficiency of secondary prevention strategies in this population.

Assessment of the adequate implementation secondary prevention strategies (i.e. pharmacological intake, smoking habit withdrawal, blood pressure, glycosylated hemoglobin, triglyceride and cholesterol levels) was performed 12 months after the index STEMI episode. The closest laboratory and blood pressure values were retrieved by means of manual review of the patient’s electronic clinical history. A 6 to 18-month timeframe from the STEMI was allowed for the obtainment of such clinical and laboratory variables, following previously reported methodology [10, 14, 15, 18–20]. As for patients who died between the 6 months and the first year after the STEMI, the latest risk factors assessment before their decease was incorporated into the study database.

Adequate control of cardiovascular risk factors was established, following current guidelines, as follows: blood pressure <140/90 mmHg; smoking abstinence for at least 6 months; total cholesterol of <200 mg/dL, LDL-cholesterol of <70 mg/dL or ≥50% lower with respect to baseline values, HDL-cholesterol >40 mg/dL and triglycerides below 150 mg/dL [18–21, 25–28]. Glycosylated hemoglobin levels of <7.5% were pursued among diabetic study patients [18–21, 26].

Our institutional guidelines and recommendations include that all patients should undergo a cardiovascular risk factors assessment during the first year after the index STEMI event and assuming that this information was available in the regional electronic health recording system, a particular risk factor was considered: i) *controlled* when the targeted goal was achieved; ii) *not controlled* when the targeted goal was not achieved; and iii) *not assessed* when no information related to the risk factor was available in the electronic health record system.

A longer follow-up study period (beyond the 12-months study period) was allowed for the assessment of mortality and cardiovascular readmission rates.

### Statistical analysis

Results are expressed as absolute and relative frequencies for categorical variables, as means and standard deviations (SD) or, when appropriate, as medians and interquartile ranges (IQR), for continuous variables. Student’s *T*-test or Man-Whitney *U* test was used for the comparison of quantitative variables and the chi-squared was used for the comparison of qualitative variables. A multinomial multivariate analysis was carried out in order to identify those clinical variables associated with an adequate control of the cardiovascular risk factors in our population. Finally, in order to assess the influence of risk factors control in the long-term prognosis of our population, a Cox proportional hazard regression model was used. Those variables associated with the outcomes of interest in the univariate analysis (*p*-value < 0.05) were included in the multivariate models, moreover we also included age, as a continuous variable, and sex. All analyses were performed using the R statistical package.

## Results

### Patient population

**Fig. 1:**
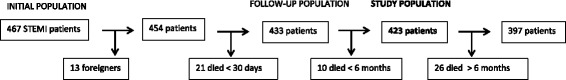
Study patient’s flow-chart

Out of the 467 STEMI patients consecutively admitted in our Institution upon activation of the *AMI code*, 13 were excluded because they were foreigners/displaced. Of the remaining 454 patients, 433 (95%) survived the first 30 days and 423 (93%) survived the first 6 months, the latter comprising our study population (Fig. 1). The percentage of missing data was null for the main variables of interest (control of cardiovascular risk factors and clinical outcomes in the follow-up) and < 5% for the covariates included in the multivariate analyses.

The clinical and demographic baseline characteristics of the 6-month surviving 423 patients are shown in Table 1. Of note, urgent reperfusion therapy was performed in 370 (87%) patients, in nearly all 363/370 (98%) by means of primary PCI. In the remaining seven patients (2%), fibrinolysis was preferred upon a presumably delayed access to urgent PCI. An excessively delayed time from the onset of symptoms until first medical attendance was the principal reason for reperfusion therapy not being carried out (53 out of the 423 patients not undergoing urgent reperfusion, 13%). The median time between first medical contact and reperfusion was 94 (69–128) minutes. The pharmacological treatment administered during the procedure and the hospital admission, as well as the in-hospital complications are summarized in Additional file 1: Table S1.

**Table 1:** Baseline characteristics of the 6-month survivors ST-elevation myocardial infarction patients included in this registry

Patient characteristics	Total (*n* = 423)	Men (*n* = 319)	Women (*n* = 104)	*p*-value
Age (years)	63 ± 13.1	60.9 ± 12.7	69.2 ± 12.4	<0.001
Current smokers	201 (47.9%)	173 (54.4%)	28 (27.5%)	<0.001
Former smokers (>1 year)	100 (23.8%)	92 (28.9%)	8 (7.8%)	<0.001
Hypertension	235 (55.5%)	165 (51.7%)	70 (67.3%)	<0.001
Dyslipidemia				0.062
Drug treatment No drug treatment	134 (32.5%) 73 (17.7%)	94 (30.3%) 62 (20%)	40 (39.2%) 11 (10.8%)	
Diabetes mellitus	112 (26%)	76 (24%)	36 (35%)	0.107
Family history^a^	39 (11%)	29 (10.5%)	10 (12.3%)	0.800
Previous ischemic heart disease	44 (10.4%)	35 (11%)	9 (8.7%)	0.626
Chronic kidney disease	14 (3.4%)	7 (2.3%)	7 (6.9%)	0.052
Anemia	33 (8.2%)	19 (6.3%)	14 (14.1%)	0.023
AMI data				
Systolic blood pressure at admission (mmHg)	130 [114–150]	130 [112–148]	140 [120–164]	0.003
Infarct location				0.713
Anterior	151 (35.7%)	112 (35.1%)	39 (37.5%)	
Inferior	231 (54.6%)	178 (55.8%)	53 (51%)	
Killip class				0.137
I	341 (83.2%)	264 (84.9%)	77 (77.8%)	
II	35 (8.1%)	22 (7.1%)	11 (11.1%)	
III	16 (3.9%)	9 (2.9%)	7 (7.1%)	
IV	21 (4.9%)	16 (5.1%)	4 (4.1%)	
LVEF	52.1 ± 12	52,5 ± 12	51 ± 12	0.283
Blood test at admission	Total (*n* = 423)	Men (*n* = 319)	Women (*n* = 104)	*p*-value
Hb (gr/dL)	14 ± 1.8	14.4 ± 1.7	12.9 ± 1.7	<0.001
GFR (mL/min/m2)	60 [60–60]	60 [60–60]	60 [60–60]	0.006
Basal glucose (mg/dL)	111 [98–136]	110 [98–133]	114 [97–148]	0.423
HbA1c (%) (in DM)	7.25 [6.60–8.53]	7.30 [6.60–8.53]	7.10 [6.60–8.08]	0.643
Total Cholesterol (mg/dL)	183 [150–214]	182 [151–212]	188 [150–219]	0.425
LDL-Cholesterol (mg/dL)	112 [84–139]	112 [85–138]	112 [82–142]	0.842
HDL-Cholesterol (mg/dL)	44 [37–53]	42 [36–50]	49 [41–60]	<0.001
Triglycerides (mg/dL)	120 [88–168]	124 [91–173]	112 [80–159]	0.061

Results are expressed as n (%), mean ± standard desviation or median [interquartile range]. *P*-value expresses the differences between men and women

*AMI* acute myocardial infarction, *LVEF* left-ventricular ejection fraction, *Hb* hemoglobin, *GFR* glomerular filtration rate, *HbA1c* Glycosylated hemoglobin, *LDL* low density lipoprotein, *HDL* high density lipoprotein

^a^Family history: early ischemic heart disease in first-degree relatives

### Cardiovascular risk factors at 1-year follow-up

Remarkably, ongoing pharmacological secondary prevention strategies was the rule among the study population, with 95% of the patients receiving statins, 90% on acetylsalicylic acid, 86% on beta-blockers and 78% on ACE inhibitors or ARBs at the end of the follow-up period.

**Fig. 2:**
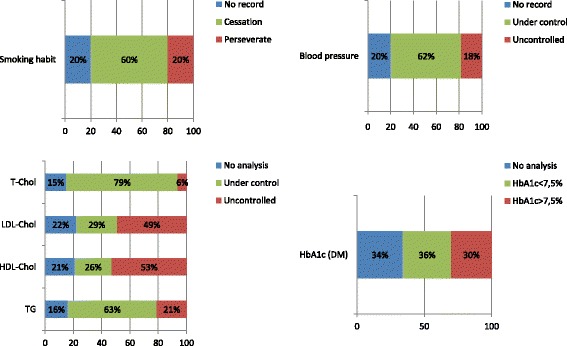
Control of cardiovascular risk factors during follow-up. In *blue* it is depicted the percentage of patients in whom a particular cardiovascular risk factor was not conveniently assessed. In *green* it is depicted the percentage of patients in whom a particular cardiovascular risk factor is under control. In *red* it is depicted the percentage of patients in whom a particular cardiovascular risk factor is out of the targeted range

Assessment of cardiovascular risk factors at 1-year follow-up is summarized in Fig. 2 and Table 2. Blood pressure and LDL-Cholesterol levels were not assessed in 84 (20%) and 93 (22%) patients, respectively (Fig. 2). Smoking habit withdrawal was not assessed in 41 out of the 201 smokers (20%) included in the study. Finally, in 38 out of the 112 diabetic patients (34%), the glycosylated hemoglobin was not available (Fig. 2). In those patients with a proper assessment of cardiovascular risk factors, a significant descent of total cholesterol (from 184 [179–188] to 155 [151–159] mg/dL, *p* < 0.001), LDL-cholesterol (112 [108–116] to 86 [82–89] mg/dL, *p* < 0.001) and triglycerides levels (from 120 [88–168] to 105 [81–150] mg/dL, *p* < 0.001) was observed. However, only 29% of patients had their LDL-cholesterol values below the targeted levels 1 year after the STEMI episode (Fig. 2). No significant changes in HDL-cholesterol levels were observed (*p* = 0.699). Nearly two-thirds of patients (263 patients, 62%) had an adequate blood pressure control and a significant descent in systolic blood pressure was observed from hospital admission to 1 year after the STEMI (133 [130–136] to 126 [124–128] mmHg, *p* < 0.001). As for diabetic patients, the glycosylated hemoglobin levels had significantly decreased as compared to baseline (from 7.9 [7.5–8.3] to 7.75 [7.3–8.2] %, *p* = 0.02), although with only one third of them (*n* = 36%) having their glycosylated hemoglobin levels below 7.5% (Fig. 2). Finally, 120 out of the 201 smokers (60%) had given up smoking at the end of the follow-up. Altogether, only 18 out of 311 (5.8%) non-diabetic and 2 out of 112 (1.8%) diabetic patients met all the secondary prevention strategy targets at the end of the 1-year follow-up period.

**Table 2:** Cardiovascular risk factors distribution at the end of the 1-year follow-up period

	Global	Men	Women	*p*-value
Systolic blood pressure (mmHg)	126 [115–137]	125 [115–136]	130 [116–137]	0.355
Total Cholesterol (mg/dL)	148 [129–175]	146 [127–167]	163 [140–191]	<0.001
LDL-Cholesterol (mg/dL)	80 [65–99]	77 [65–93]	90 [67–112]	0.005
HDL-Cholesterol (mg/dL)	44 [38–52]	44 [36–50]	49 [42–57]	<0.001
Triglycerides (mg/dL)	105 [81–150]	102 [81–146]	116 [82–158]	0.245
HbA1c (%) (in DM)	7.75 ± 1.84	7.78 ± 1.91	7.68 ± 1.71	0.824

Results are expressed as n (%), mean ± standard desviation or median [interquartile range]. *P*-value test differences between men and women

*LDL* low density lipoprotein, *HDL* high density lipoprotein, *HbA1c* glycosylated hemoglobin, DM (diabetes mellitus)

No clinical variables were associated to an inadequate control of cardiovascular risk factors, with the exception of age, which was associated with a lower probability of inadequate control of HDL-cholesterol (OR = 0.96; 95% CI: 0.94–0.99; *p* = 0.005) and triglycerides (OR = 0.96; 95% CI: 0.93–0.99; *p* = 0.003). Age was also associated with lower probability of smoking habit continuation (OR = 0.93; 95% CI: 0.89–0.98; *p* = 0.004).

### Impact of clinical variables and risk factor control on long-term cardiovascular outcome

Differences in baseline clinical characteristics in patients with events versus without events (cardiovascular readmission or death from any cause) during follow-up are shown in Table 3.

**Table 3:** Baseline clinical characteristics of patients with versus without events during follow-up

Patient characteristics	Event (*n* = 61)	No event (*n* = 362)	*p*-value
Age (years)	67 ± 15	63 ± 13	0.047
Current smokers	25 (%)	168 (46%)	0.295
Hypertension	44 (72.1%)	186 (51.4%)	0.016
Dyslipidemia	32 (52.5%)	170 (47%)	0.356
Diabetes mellitus	16 (26.2%)	72 (19.9%)	0.273
Family history*	6 (9.8%)	31 (8.6%)	0.946
Chronic kidney disease	3 (4.9%)	11 (3%)	0.448
Anemia	10 (16.4%)	23 (6.4%)	0.018
AMI data^a^	Event (*n* = 61)	No event (*n* = 362)	*p*-value
Infarct location			0.08
Anterior	29 (47.5%)	116 (32%)	
Inferior	28 (45.9%)	196 (54%)	
Killip class			<0.001
I	37 (60.6%)	300 (82.9%)	
II	9 (14.8%)	30 (8.3%)	
III	9 (14.8%)	13 (3.6%)	
IV	6 (9.8%)	19 (5.2%)	
LVEF^a^	46.3 ± 12.6	53.1 ± 11.3	<0.001

Event included cardiovascular readmission (due to heart failure, acute coronary syndrome or stroke) or death from any cause. Results are expressed as n (%) or mean ± standard deviation

^a^ *AMI* acute myocardial infarction, *LVEF* left-ventricular ejection fraction; Family history: early ischemic heart disease in first-degree relatives

The median follow-up study period was 20 (11–30) months. A total of 26 patients (6.1%) died during follow-up and 42 (9.9%) were readmitted due to cardiovascular causes. Hospital readmission was due to heart failure in 21 patients, due to acute coronary syndrome in 14, and due to stroke in 7. Population who were readmitted or died during follow-up were older, had a higher prevalence of systemic hypertension and anemia, scored higher in the Killip classification and had a lower left ventricular ejection fraction at hospital-discharge (Table 3). In the multivariate analysis, hypertension, Killip class III-IV, anemia and depressed left ventricular ejection fraction were associated with both, mortality and cardiovascular readmissions (Table 4).

**Table 4:** Clinical variables associated with mortality and cardiovascular readmission in multivariate analysis

Mortality	HR [CI 95%]	*p*-value
Age	1.02 [0.98–1.06]	0.24
Women	0.89 [0.34–2.30]	0.81
Hypertension	3.73 [1.18–11.82]	0.03
Anemia	3.82 [1.47–9.92]	0.03
Killip class III-IV	5.40 [2.05–14.24]	<0.001
LVEF > 45%	0.22 [0.09–0.55]	<0.001
Cardiovascular readmission	HR [CI 95%]	*p*-value
Age	1.01 [0.98–1.04]	0.42
Women	1.34 [0.70–2.59]	0.38
Hypertension	2.72 [1.26–5.87]	0.01
Anemia	1.39 [0.59–3.29]	0.45
Killip class III-IV	3.56 [1.49–8.49]	<0.001
LVEF > 45%	0.37 [0.20–0.69]	<0.001

*LVEF* left-ventricular ejection fraction, *HR* hazard ratio, *CI* confidence intervals

Regarding the association between inadequate assessment or control of the conventional cardiovascular risk factors and long-term cardiovascular mortality and readmission rates, the lack of assessment of LDL- cholesterol and HDL-cholesterol were significantly associated with a worse cardiovascular outcome. No other differences were noted for other *not-assessed or not controlled* variables (Table 5).

**Table 5:** Age, sex, hypertension, anemia and Killip adjusted *Hazard ratio* for mortality or cardiovascular readmission for those patients with no assessed or with no controlled cardiovascular risk factors (respect to patients with controlled risk factors)

		HR [CI 95%]	*p*-value
Total cholesterol	Not assessed Not controlled	1.47 [0.74–2.93] 0.18 [0.02–1.36]	0.28 0.10
LDL-cholesterol	Not assessed Not controlled	2.57 [1.26–5.24] 0.89 [0.44–1.81]	0.01 0.76
HDL-cholesterol	Not assessed Not controlled	2.88 [1.54–5.39] 1.26 [0.61–2.61]	<0.001 0.53
Triglycerides	Not assessed Not controlled	1.80 [0.90–3.59] 1.12 [0.56–2.21]	0.09 0.75
HbA1c	Not assessed Not controlled	0.80 [0.19–3.31] 0.48 [0.90–2.66]	0.76 0.40
Blood pressure	Not assessed Not controlled	1.04 [0.51–2.12] 0.58 [0.25–1.32]	0.92 0.19
Smoking	Not assessed Not controlled	0.73 [0.38–1.40] 2.05 [0.83–5.05]	0.35 0.12

*HbA1c* glycosylated hemoglobin

When only considering patients undergoing primary PCI (*n* = 363) or any reperfusion therapy (*n* = 370), the long-term adherence to secondary prevention strategies and its clinical impact were neither significantly different.

## Discussion

In this study it is demonstrated that the long-term efficiency of conventional secondary prevention strategies after a STEMI episode in the *AMI code* era (a therapeutic strategy which enhances a rapid and widespread access to urgent PCI) is manifestly suboptimal. Impressively, less than 6% of diabetic or non-diabetic patients met all the secondary prevention strategy targets at the end of the follow-up period.

Additionally, we have noted that an inadequate assessment of the LDL- and HDL-cholesterol levels is significantly associated with a less favorable long-term cardiovascular outcome after STEMI. Although no other prognostic impact of an adequate/inadequate risk factors control was demonstrated in our series, efficacy of secondary prevention strategies after a STEMI episode has been widely proven elsewhere [1–4, 9, 14–20]. It is suggested by this that an inadequate control of conventional cardiovascular risk factors after a STEMI episode may, at least partially, counterbalance the demonstrated prognostic benefits of current myocardial reperfusion strategies, such as urgent PCI [6–8, 10, 13]. Therefore, further efforts should be driven in order to enhance a more adequate accomplishment of all secondary prevention strategies after a STEMI, since these efforts should have a positive impact on the cardiovascular outcome in such patients in the long-term.

Not surprisingly, patients presenting with cardiovascular readmission or death from any cause during follow-up were older and had a higher prevalence of systemic hypertension, anemia, scored higher in Killip class classification and had lower ejection fraction. All of such conventional risk factors except for age still remain as robust predictors of a poorer cardiovascular outcome after a STEMI episode.

### Long-term assessment and control of cardiovascular risk factors after STEMI

Notably, around one-sixth of our patient population did not undergo a blood test control after the index STEMI episode, with one third of the diabetic population not having glycosylated hemoglobin determinations. These figures are important and highlight the limitations of the implementation of current secondary prevention strategies among these patients. Patient education and physician awareness of this situation are some of the elements that could be implemented to decrease these poor results [21].

According to the current European clinical practice guidelines of secondary prevention, the current objective for LDL-cholesterol is to achieve a concentration inferior to 70 mg/dL or a reduction greater than 50% from the basal LDL-cholesterol levels [18, 19]. In our study, despite a high prescription rate of statins during follow-up, only one third of our patients reached the targeted levels. The EUROASPIRE IV (carried out in several European centers) and ADVANCE studies were undertaken in patients with stable coronary artery disease, and neither showed encouraging results [25, 26].

Previously reported European multicenter registers carried out over nearly two decades in a population of patients with CAD demonstrated that only half of the patients have their blood pressure under control despite of the high prescription rate of ACE inhibitors and beta blockers [25, 28]. These results are comparable with our observations, with only 62% of the patients having an adequate blood pressure control, besides the 20% percentage of the population with blood pressure control not being adequately assessed. Although a significant decrease in the systolic blood pressure values was established during follow-up as compared to the initial value upon hospital admission for the index STEMI episode (*p* < 0.001), this data has to be analyzed cautiously. The initial blood pressure value registered during an acute coronary event is not necessarily representative of the baseline patient’s blood pressure control. Furthermore, the prognostic value of high systolic blood pressure values at admission in the setting of an acute STEMI remains controversial [29–32].

The observed suboptimal metabolic control of diabetic patients is in the line of previous observations reported in series of diabetic patients undergoing by-pass surgery or PCI, with around one-half of diabetic patients having inadequate and/or insufficient long-term glycosylated hemoglobin levels control [27].

Smoking cessation after STEMI is associated with improved survival rates [14, 33]. Our study confirms an unacceptably high rate of smoking habit continuation, thus suggesting that further counseling strategies pursuing smoking habit withdrawal should be rendered in this setting.

### Myocardial invasive reperfusion and prognosis

The implementation of the *AMI code* program has increased the access to urgent myocardial reperfusion to an 87% of STEMI patients, nearly all of them through PCI [8]. This increase in the use of urgent reperfusion strategies is similar to that observed in another national multicenter register, with a reperfusion rate of 85.7% of patients, the difference being that, in the latter, one third of reperfusions were performed by means of fibrinolysis [3].

In our study, the 30-day mortality rate (5%) was similar to that observed in other national and international surveys in the PCI era [10, 34–39]. However, 6.1% of the STEMI 6-months survivors had deceased at the end of the 20-months (median) follow-up period, with an additional 9.9% of cardiovascular readmissions, percentages that (although low) have to be still considered suboptimal and in line with previous national series in which widespread use of urgent PCI was not yet the rule [3].

Our findings should make us reconsider the actual therapeutic yield of urgent myocardial reperfusion strategies in the setting of a STEMI: while the implementation of assistance networks (such as the *AMI code* program) has ameliorated the acute management of STEMI, the potentially beneficial impact of such strategies may be jeopardized by a suboptimal long-term implementation of the secondary prevention strategies. In the view of our results, we believe further efforts devoted to promote the cessation of smoking, periodical blood testing and blood pressure control, and to more intensively maintain LDL-cholesterol, glycosylated hemoglobin and blood pressure below the targeted levels should be undertaken. The importance of an adequate risk factors control has been highlighted also for patients with stable coronary disease, and may be specially manifest among patients with low ejection fraction and (like in our series) with a prior history of myocardial infarction [40].

#### Study limitations

The lack of association between cardiovascular risk factors control and cardiovascular outcome in our series is probably due to the low total number of cardiovascular events, the relatively short follow-up time and the limited sample size, reducing the statistical power of our analyses. Otherwise, dose of statins and other medication, adherence to prescribed treatment, healthy lifestyles such as diet, physical exercise, and weight control, potentially influencing the clinical outcome after a STEMI episode, were not assessed in our series due to limitations inherent to the use of the regional electronic health recording system. Finally, our single-center study may not be representative of the Spanish/European population of STEMI patients. However, its prospective nature and the consecutive inclusion appear to bestow a representative sample of real life.

## Conclusions

Although implementation of the *AMI code* program has resulted in a widespread access to urgent myocardial reperfusion by means of PCI, the secondary prevention strategies after a STEMI episode are still far from being optimal, thus potentially counterbalancing the prognostic benefits of this therapy. Additional efforts to optimize the assessment and control of conventional cardiovascular risk factors should be implemented.

## Additional file

Additional file 1:
**Table S1.** Complications and in-hospital treatment. (DOCX 16 kb)
