# Supplementary material for: Secondary prevention strategies after an acute ST-segment elevation myocardial infarction in the AMI code era: beyond myocardial mechanical reperfusion
Source: BMC Cardiovasc Disord. 2017 Feb 7;17:54. doi: 10.1186/s12872-017-0493-6 (PMC5297147; doi:10.1186/s12872-017-0493-6)
Supplement: Additional file 1: — Table S1. Complications and in-hospital treatment. (DOCX 16 kb) [file 12872_2017_493_MOESM1_ESM.docx]

**Table S1. Complications and in-hospital treatment.**

| **TABLE 1.** | | | | |
| --- | --- | --- | --- | --- |
| **COMPLICATIONS** | **Total (n=423)** | **Men (n=319)** | **Women (n=104)** | ***P*-value** |
| **Malignant arrhytmias (VT/VF)** | 24 (5.7%) | 21 (6.6%) | 3 (2.9%) | 0.17 |
| **Atrioventricular block** | 23 (5.4%) | 16 (5%) | 7 (6.7%) | 0.67 |
| **Temporary pacemaker** | 11 (2.6%) | 7(2.2%) | 4 (3.8%) | 0.47 |
| **Supraventricular tachycardia** | **19 (4.5%)** | **9 (2.8%)** | **10 (9.6%)** | **0.01** |
| Major bleeding | 3 (0.7%) | 2 (0.6%) | 1 (1%) | 0.43 |
| **Right ventricular infarction** | 15 (3.5%) | 11 (3.4%) | 4 (3.8%) | 0.77 |
| **TREATMENT** | **Total (n = 423)** | **Men (n = 319)** | **Women (n = 104)** | ***P*-value** |
| **A**cetylsalicylic acid | 419 (99.1%) | 316 (99.1%) | 103 (99%) | 0.43 |
| **Clopidogrel** | 361 (85.3%) | 268 (84%) | 93 (89.4%) | 0.28 |
| **Prasugrel** | 12 (2.8%) | 11 (3.4%) | 1 (1%) | 0.47 |
| **Ticagrelor** | 76 (18%) | 65 (20.4%) | 11 (10.6%) | 0.11 |
| **Anti-GPIIb/IIIa** | 58 (13.7%) | 43 (13.5%) | 15 (14.4%) | 0.99 |
| Sodium **heparin/LMWH** | 395 (93.4%) | 299 (93.7%) | 96 (92.3%) | 1 |
| **Coumarins** | 4 (0.9%) | 2 (0.6%) | 2 (1.9%) | 0.25 |
| **ACEi/ARB** | 328 (77.5%) | 243 (76.2%) | 85 (81.7%) | 0.57 |
| **Beta Blockers** | 349 (82.5%) | 263 (82.4%) | 86 (82.7%) | 1 |
| **Aldosterone antagonists** | 70 (16.5%) | 50 (16.7%) | 20 (19.2%) | 0.55 |
| **Nitrates** | 148 (35%) | 104 (32.6%) | 44 (42.3%) | 0.11 |
| **Diuretics** | **95 (22.5%)** | **60 (18.8%)** | **35 (33.7%)** | **0.003** |
| **Statins** | 416 (98.3%) | 312 (97.8%) | 104 (100%) | 1 |
| **Insuline /OHAs** | 115 (27.2%) | 82 (25.7%) | 33 (31.7%) | 0.27 |
| **Inotropic** | 28 (6.6%) | 22 (6.9%) | 6 (5.8%) | 0.86 |
| **Vasopressor** | 21 (5%) | 16 (5%) | 5 (4.8%) | 1 |

Results are expressed as n(%), average ± standard desviation or median [interquartile range].

VT=ventricular tachycardia; VF=ventricular fibrillation; Anti-GPIIb/IIIa= Glycoprotein IIb**/**IIIa inhibidors; LMWH=low**-**molecular**-**weight heparin; ACEi=angiotensin-converting-enzyme inhibitor; ARB=[Angiotensin receptor blocker](https://en.wikipedia.org/wiki/Angiotensin_receptor_blocker); OHAs=Oral hypoglycemic agents.
